# Azithromycin to prevent acute lower respiratory infections among Australian and New Zealand First Nations and Timorese children (PETAL trial): study protocol for a multicentre, international, double-blind, randomised controlled trial

**DOI:** 10.1136/bmjopen-2024-097455

**Published:** 2025-02-05

**Authors:** Gabrielle B McCallum, Catherine A Byrnes, Peter S Morris, Keith Grimwood, Robyn L. Marsh, Mark D Chatfield, Emily R Bowden, Kobi L Schutz, Nevio Sarmento, Nicholas Fancourt, Joshua Francis, Yuejen Zhao, Adriano Vieira, Kim M Hare, Dennis Bonney, Adrian Trenholme, Shirley Lawrence, Felicity Marwick, Bronwyn Karvonen, Carolyn Maclennan, Christine Connors, Heidi Smith-Vaughan, Milena Santos Lay, Endang Soares da Silva, Anne B Chang

**Affiliations:** 1 Child and Maternal Health, Menzies School of Health Research, Darwin, Northern Territory, Australia; 2 Department of Pediatrics, Starship Children's Health, Auckland, Auckland, New Zealand; 3 The University of Auckland Department of Paediatrics Child and Youth Health, Auckland, Auckland, New Zealand; 4 School of Medicine and Dentistry, Griffith University, Southport, Queensland, Australia; 5 University of Tasmania School of Health Sciences, Launceston, Tasmania, Australia; 6 Faculty of Health, Medicine and Behavioural Sciences, University of Queensland, Kedron, Queensland, Australia; 7 Global and Tropical Health, Menzies School of Health Research, Casuarina, Northern Territory, Australia; 8 The University of Sydney Faculty of Medicine and Health, Sydney, New South Wales, Australia; 9 Health Statistics and Informatics, Northern Territory Department of Health, Casuarina, Northern Territory, Australia; 10 Department of Paediatrics, Royal Darwin Hospital Department of Maternal and Child Health, Darwin, Northern Territory, Australia; 11 Department of Paediatrics, Middlemore Hospital, Auckland, Auckland, New Zealand; 12 Northern Territory Department of Health, Casuarina, Northern Territory, Australia; 13 School Nurse, St Francis of the Fields Primary School, Strathfieldsaye, Victoria, Australia; 14 Top End Health Services, Northern Territory Department of Health, Casuarina, Northern Territory, Australia; 15 Department of Paediatrics, Guido Valadares National Hospital, Dili, Timor-Leste; 16 Partnership for Human Development, Dili, Timor-Leste; 17 The Australian Centre for Health Services Innovations, Queensland University of Technology, Brisbane, Queensland, Australia

**Keywords:** Respiratory infections, Antibiotics, Child, Paediatric thoracic medicine, Randomized Controlled Trial

## Abstract

**Introduction:**

Acute lower respiratory infections (ALRIs) remain the leading causes of repeated hospitalisations among young disadvantaged Australian and New Zealand First Nations and Timorese children. Severe (hospitalised) and recurrent ALRIs in the first years of life are associated with future chronic lung diseases (eg, bronchiectasis) and impaired lung function. Despite the high burden and long-term consequences of severe ALRIs, clinical, evidence-based and feasible interventions (other than vaccine programmes) that reduce ALRI hospitalisations in children are limited. This randomised controlled trial (RCT) will address this unmet need by trialling a commonly prescribed macrolide antibiotic (azithromycin) for 6–12 months. Long-term azithromycin was chosen as it reduces ALRI rates by 50% in Australian and New Zealand First Nations children with chronic suppurative lung disease or bronchiectasis. The aim of this multicentre, international, double-blind, placebo-containing RCT is to determine whether 6–12 months of weekly azithromycin administered to Australian and New Zealand First Nations and Timorese children after their hospitalisation with an ALRI reduces subsequent ALRIs compared with placebo. Our primary hypothesis is that children receiving long-term azithromycin will have fewer medically attended ALRIs over the intervention period than those receiving placebo.

**Methods and analysis:**

We will recruit 160 Australian and New Zealand First Nations and Timorese children aged <2 years to a parallel, superiority RCT across four hospitals from three countries (Australia, New Zealand and Timor-Leste). The primary outcome is the rate of medically attended ALRIs during the intervention period. The secondary outcomes are the rates and proportions of children with ALRI-related hospitalisation, chronic symptoms/signs suggestive of underlying chronic suppurative lung disease or bronchiectasis, serious adverse events, and antimicrobial resistance in the upper airways, and cost-effectiveness analyses.

**Ethics and dissemination:**

The Human Research Ethics Committees of the Northern Territory Department of Health and Menzies School of Health Research (Australia), Health and Disability Ethics Committee (New Zealand) and the Institute National of Health-Research Technical Committee (Timor-Leste) approved this study. The study outcomes will be disseminated to academic and medical communities via international peer-reviewed journals and conference presentations, and findings reported to health departments and consumer-based health organisations.

**Clinical trial registration:**

Australia New Zealand Clinical Trial Registry ACTRN12619000456156.

**STRENGTHS AND LIMITATIONS OF THIS STUDY**

- The randomised controlled trial is multicentre, involving three countries.
- Standardised methods from our previous azithromycin trials will be used.
- Outcome measures are clinically relevant and clearly defined.
- The trial is powered to address the primary and secondary objectives.
- A potential limitation that may affect attrition is the duration of follow-up for 24 months.

## Introduction

 Acute lower respiratory infections (ALRIs (including bronchiolitis and pneumonia)), remain the most common global cause of child morbidity and mortality beyond the neonatal period.^1^ In 2021, there were an estimated 37.8 million ALRIs worldwide resulting in 502 000 deaths in children aged <5 years.^2^ The greatest burden was in children from low- and middle-income countries (LMICs) where 98.4% of ALRIs and 99.8% of deaths occurred.^2^ For example, in Timor-Leste, a LMIC, the ALRI incidence and its associated mortality in 2021 was 48 and 1.31/1000 children aged <5 years, respectively, compared with 11 and 0.3/1000 children aged <5 years in high-income countries, respectively.^2^ Nevertheless, there is also a high disease burden in disadvantaged First Nations children in high-income countries, especially during infancy where ALRI hospitalisation rates in the USA,^3^ Australia^4^ and New Zealand Māori and Pacific infants are 116, 427, 131, and 187/1000 infants aged <12 months, respectively.^5^

Severe (hospitalised) and recurrent ALRIs in the first years of life when the lungs are developing and vulnerable to injury are associated with future impaired lung health,^6^ reduced lung function trajectories^7 8^ and subsequent increased risk of chronic lung diseases (eg, bronchiectasis and chronic obstructive pulmonary disease (COPD)).^9^,^11^ Aboriginal and Torres Strait Islanders, and New Zealand Māori, and Pacific Islanders (henceforth referred to as First Nations), and Timorese children are at high risk of recurrent ALRIs,^4 6^ and in First Nations people chronic lung disease.^6 12^ Despite the high burden and long-term consequences of severe ALRIs, there are limited, clinically feasible strategies except national immunisation programmes, which include influenza and/or pneumococcal and *Haemophilus influenzae* type b conjugate vaccines^13^ that reduce the risk of both ALRI hospitalisations and recurrent ALRIs in young children.

The importance of reducing recurrent ALRIs among children hospitalised previously is reflected in data from studies involving children from settings where the burden of acute and chronic lung disease is particularly high.^2^ A prospective cohort study of First Nations children with either chronic suppurative lung disease (CSLD) or radiographic-confirmed bronchiectasis from Alaska, Australia and New Zealand found at baseline these children had an earlier onset and higher frequency of ALRIs than their local First Nations populations.^10^ Our previous RCTs^14 15^ that included 162 Northern Territory First Nations children aged <2 years hospitalised with bronchiolitis reported 41 children (25%) were rehospitalised with a respiratory illness within 6 months of hospital discharge. Furthermore, 30/157 (19%) had developed bronchiectasis by 13 months (IQR, 7–18) post-hospitalisation.^16^ In New Zealand, a study among 94 First Nations children aged <2 years found 74% had ongoing respiratory morbidity (ie, cough, chest X-ray changes) 12 months after an ALRI hospitalisation.^17^ This included three children diagnosed with bronchiectasis post-hospitalisation by chest high-resolution CT (HRCT) scans undertaken for clinical reasons.

One feasible, but untested, intervention to reduce recurrent ALRIs is prescribing long-term oral azithromycin, an antimicrobial agent with immunomodulatory and anti-inflammatory properties.^18^ Azithromycin is well tolerated and its half-life of 70 hours allows infrequent dosing regimens (eg, once per week) that reduces the burden on families. Our previous randomised controlled trial (RCT) among First Nations children from Australia and New Zealand with CSLD and bronchiectasis found weekly azithromycin for up to 24 months halved respiratory exacerbation rates while receiving the active treatment.^19^ Children who received azithromycin were also less likely to be hospitalised for respiratory illness during this period.^19^ RCTs in adults^20^ and subsequent trials by other groups that included children also found similar effects. For example, long-term azithromycin for 6 months reduced exacerbation rates by 55% among children and young adults with primary ciliary dyskinesia^21^ and also by a similar margin in Southern African children with HIV-associated chronic lung disease.^22^ Nevertheless, despite these positive results, concerns remain over the selection of macrolide-resistant pathogens associated with long-term azithromycin.^23 24^

In contrast, data for treating virus-associated ALRIs are less convincing. We previously found azithromycin given for up to 3 weeks to treat a single episode of bronchiolitis in Australian and New Zealand First Nations children did not improve short-term outcomes (hospitalisation or wheeze by 6 months).^14 15^ A recent systematic review and meta-analysis involving seven RCTs reported azithromycin did not provide any meaningful clinical benefit in children aged <2 years hospitalised with bronchiolitis and did not reduce subsequent wheezing episodes.^25^

Despite the current lack of evidence for long-term azithromycin in children hospitalised previously for an ALRI, it is often prescribed in the Northern Territory of Australia. For example, our prospective study of children undergoing evaluation for chronic cough at Royal Darwin Hospital revealed 27% of 304 children (88% First Nations, median age 2.3 years (IQR 1.5–3.8)) had received long-term azithromycin before bronchiectasis was confirmed by chest HRCT scans.^26^ Furthermore, it is unknown how many children are prescribed azithromycin routinely in primary healthcare settings for a chronic cough without referral for appropriate investigations. In our follow-up study of 162 Northern Territory children hospitalised previously for bronchiolitis,^16^ 43 (26%) were receiving long-term azithromycin prescribed by their general paediatrician for recurrent ALRIs.

In summary, young children in disadvantaged First Nations and LMIC populations have a large ALRI disease burden. This is when lungs are most susceptible to injury, leading to future impaired lung growth and function and subsequent chronic lung diseases. An intervention to prevent ALRIs during this critical phase of lung development may have both short- and long-term benefits. Following its success in clinical trials, long-term (>6 months) azithromycin is now recommended for preventing exacerbations in bronchiectasis.^27^ Despite lacking robust clinical data, there is also frequent ‘off-label’ use of azithromycin for chronic or recurrent respiratory symptoms, including ALRIs, in some communities noted for their high risk of CSLD and bronchiectasis. Taken together, there is an urgent need for high-quality RCTs to determine whether long-term azithromycin in young children following an ALRI hospitalisation is safe and efficacious at preventing further ALRIs when living in settings where a high burden of chronic lung disease and risk for future lung impairment exists.

## Aims and hypotheses

The primary aim of this RCT is to determine whether 6–12 months of weekly azithromycin (compared with placebo) following hospitalisation for an ALRI reduces the rate of subsequent episodes in First Nations and Timorese children aged <2 years. Our primary hypothesis is that children receiving long-term azithromycin will have fewer medically attended ALRIs over the intervention period than those receiving placebo.

Our secondary aims are to determine the effect of 6–12 months of weekly azithromycin (compared with placebo) on the rates and proportions of participants with ALRI-related hospitalisations, adverse events, nasopharyngeal carriage and antimicrobial resistance of respiratory pathogens and subsequent chronic respiratory symptoms/signs suggestive of underlying CSLD or bronchiectasis at 24 months. We will also assess the cost-effectiveness of the intervention by collecting healthcare-related costs and calculating the incremental cost-effectiveness ratio (ICER) on the potential benefits of azithromycin on hospitalisation, medical evacuations and other associated costs to health services compared with placebo.

Our secondary hypotheses are that 6–12 months of weekly azithromycin will result in reduced hospitalised ALRI episodes and reduced chronic respiratory symptoms/signs suggestive of underlying CSLD or bronchiectasis at 24 months, will be safe and will provide health economic benefits to health services, but with increased antimicrobial resistance in nasopharyngeal respiratory pathogens.

## Methods and analysis

### Design

**Figure 1:**
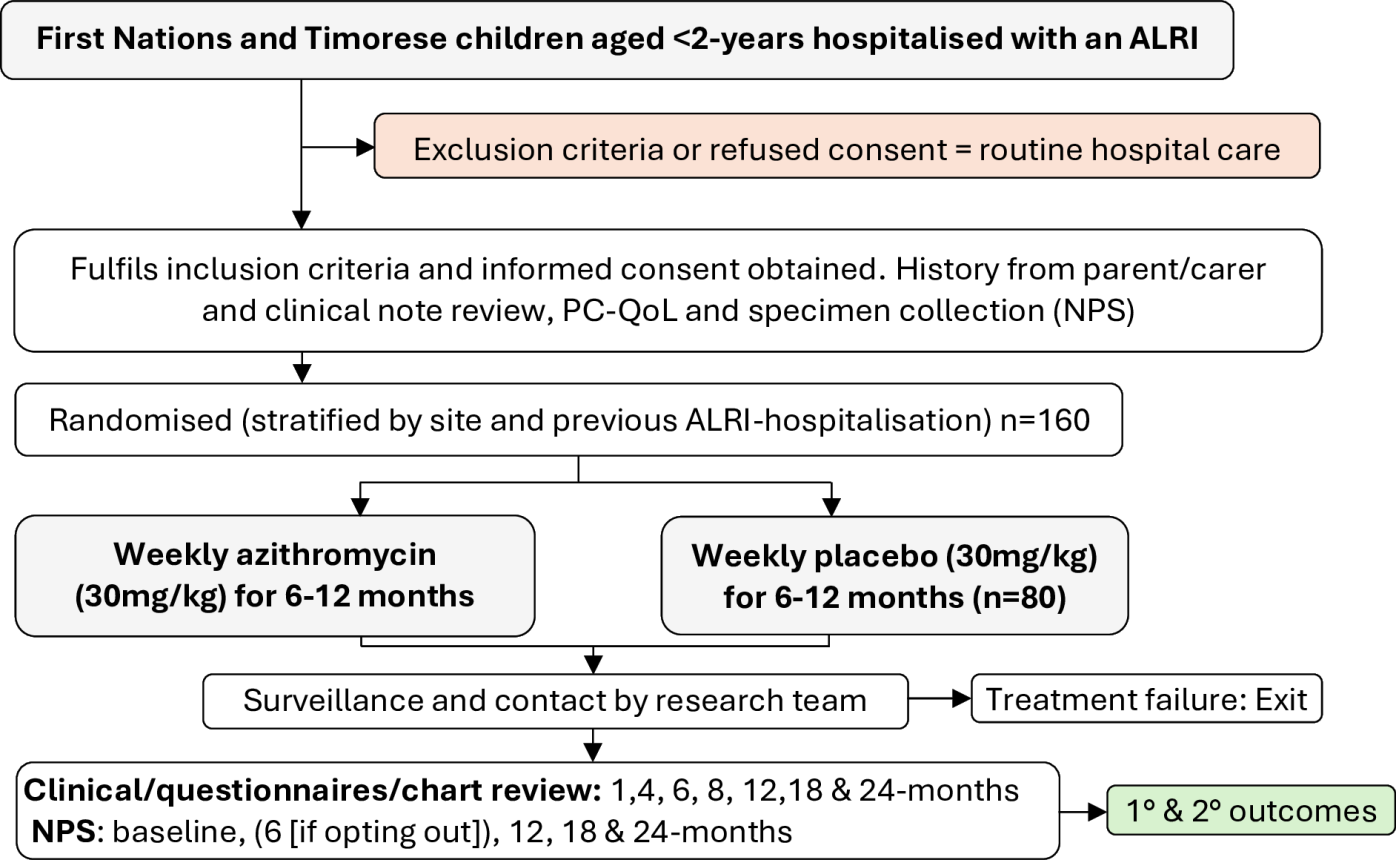
Trial design. ALRI, acute lower respiratory infection; NPS, nasopharyngeal swab; PC-QoL, parent-cough quality of life.

The azithromycin to **‘**Prevent rEcurrenT Acute Lower Respiratory Infections’ (PETAL) trial is a parallel (1:1), double-blind, placebo-controlled, multicentre, international superiority RCT (figure 1). It aims to determine whether 6–12 months of azithromycin following ALRI hospitalisation reduces subsequent ALRIs and improves other clinical and economic outcomes. The first participant was randomised in October 2020, and we anticipate that recruitment will be completed by February 2025.

### Study settings and participants

#### Study sites

The RCT is being conducted in four hospitals across three countries: Australia, New Zealand and Timor-Leste. The Australian site is Royal Darwin Hospital (Darwin), the New Zealand sites are Starship Children’s Hospital (Auckland) and Kidz First Hospital (Auckland) and the Timor-Leste site is Guido Valadares National Hospital (Dili).

#### Inclusion criteria

(1) First Nations (Australian Aboriginal and/or Torres Strait Islander; or New Zealand Māori and/or Pacific Islander) or Timorese children aged <2 years; (2) hospitalised with an ALRI (bronchiolitis or pneumonia) and resident of the site hospitals catchment areas (in Darwin this includes two remote communities within the Northern Territory); and (3) requiring during their illness (including during hospital transfer or in the community) oxygen, respiratory or fluid (nasogastric or intravenous) support OR had an ALRI-related hospitalisation within the previous 3 months. In addition, New Zealand children are to be aged ≥3 months, a condition required by the local human research ethics committee (HREC).

#### Exclusion criteria

(1) Known chronic lung disease (eg, cystic fibrosis, radiographic-confirmed bronchiectasis, chronic neonatal lung disease); (2) receiving regular azithromycin (within the last 4 weeks); (3) macrolide contraindicated (eg, known liver dysfunction, hypersensitivity); (4) primary carer lacks a mobile phone and/or is unable to attend follow-up clinical visits over the next 24 months; or (5) participation in another respiratory-related RCT.

#### Recruitment

A good clinical practice (GCP)-trained researcher visits the paediatric wards of site hospitals daily (Monday to Friday) to identify potential participants. They approach the parents/guardian of the children fulfilling the eligibility criteria and explain the RCT, including its risks/benefits, using a plain language information booklet (flipchart-based). When required, interpreters are used. After the study is explained and the parents/guardian agrees to their child’s participation, written informed consent is requested from the parents/guardian (see online supplemental file). At enrolment, parents/guardian was also informed their child could opt out of taking the trial medication after 6 rather than 12 months. This was in response to some parents/guardians articulating concerns over the study duration. All participants are managed clinically according to standardised local protocols already in place at site hospitals.

#### Trial medications

Azithromycin provided as oral powder for suspension (200 mg/5 mL) is administered once per week for 6–12 months. Doses are weight based at 30 mg/kg/week. Equivalent placebo powder for suspension matches the active medication in taste and appearance. The Menzies School of Health Research (Menzies) purchased the trial medications (azithromycin and its corresponding placebo) from the Institute of Drug Technology (Melbourne, Australia), as done previously.^14 19 28^ Trial medications are shipped to sites by trial logistics companies.

#### Randomisation, allocation concealment and blinding

Randomisation is undertaken by each site’s pharmacy representative who allocates participants to a treatment according to a randomisation sequence. Each site has two randomisation sequences; one stratified by site and the other by whether the child has had a prior ALRI hospitalisation or not. An opaque envelope with the corresponding respective randomisation number is then opened to reveal treatment allocation as one of eight bottle letters (A–H), four allocated to azithromycin and four to placebo. The randomisation sequences were generated by an independent statistician using permuted blocks.

The first dose of trial medication is given in hospital and then dispensed weekly by either a member of the research team, primary health clinic staff or family member (whichever is appropriate) for each site. Adherence is monitored by the research team in weekly medication logs based on parent reports. People masked to the treatment allocation are those (1) receiving the treatment, (2) administering the treatment, (3) assessing the outcomes and (4) analysing the data.

#### Concomitant medications

If clinically indicated, participants may have non-macrolide antibiotics for respiratory or other infections while receiving trial medications. These data are captured on standardised data collection forms when undertaking medical chart reviews.

### Data collection, monitoring and follow-up details

Data are collected by GCP-trained researchers using standardised data collection forms similar to our previous RCTs.^14 15 28^ At enrolment, sociodemographic, clinical and economic data and nasopharyngeal swabs (where possible) are collected (table 1). Sociodemographic details include age, sex, anthropometrics, family and household size, exposure to tobacco smoking (mother and household), breastfeeding, immunisation history, medications (prior to and during hospitalisation), current and previous ALRI history, comorbidities and the eight-item parent-cough specific quality of life (PC-QoL) score.^29 30^ Clinical data include respiratory symptoms/signs, investigations and treatments before and during hospitalisation. These details are obtained either by the parent/guardians and/or review of the child’s medical records.

**Table 1:** Schedule of visits and contact points

		Month					
	Baseline (hospital)	1	4	8*	12*	18	24
Screening for eligibility	√						
Written informed consent	√						
Randomisation	√						
First dose of study medication	√						
Medical history	√						
Medical chart review	√	√	√	√	√	√	√
Clinical assessment _(where possible)_	√	√	√	√	√	√	√
PC-QoL	√	√	√	√	√	√	√
NPS†	√				√	√	√
Semistructured interview							√

* Will instead be done at 6 months for children opting out of the intervention at the 6-month time-point.

† For children living in remote locations in the unlikely event where the research team are physically unable to visit and/or other avenues have been exhausted, specimen collection will not occur in these instances.

NPSnasopharyngeal swabPC-QoLparent-cough quality of life

Medication adherence and adverse events (diarrhoea, vomiting, rash, nausea) logs are maintained either by the parents/guardian or research staff during the intervention period.

Participants are followed up clinically, including obtaining PC-QoL scores, by the research team at 1, 4, 8, 12, 18 and 24 months. Participants, whose families opt out of taking the trial medication at 6 months because it was becoming a burden on family life, are clinically reviewed at 6 months rather than at 8 months. Participants will then either leave the study or remain within the study to be followed up until 24 months. Hospital and community medical charts (electronic and paper-based) will be reviewed at each visit for ALRIs and chronic respiratory symptoms/signs treated by the child’s local doctor that suggest underlying CSLD, while evidence of bronchiectasis at 24 months will be established by reviewing hospital records for chest HRCT scan and bronchoscopy results.

Where participants have moved communities or attended multiple primary healthcare facilities during their 24-month involvement with the study, we will use our established methods from previous RCTs (described above), where missing data were <10%.^14 19^

Exit criteria include consent withdrawn by the parents/guardian, and/or intolerance to trial medication determined by study site doctors or the independent data monitoring committee (iDMC). Unblinding if necessary, will be done by site pharmacy or independent representative.

### Study definitions

#### ALRI

A medically attended episode of acute respiratory illness (ie, acute cough, dyspnoea, tachypnoea, new abnormal chest examination findings with or without fever or radiographic findings treated by primary healthcare or hospital staff). Clinic visits within 2 weeks of each other are considered part of the same ALRI.^19^

#### Bronchiectasis

Persistent or recurrent chronic (>4 weeks) wet or productive cough, often with other respiratory symptoms/signs (eg, breathlessness or auscultatory crackles), and frequent respiratory exacerbations (or ALRIs) with variable responses to antibiotics AND chest HRCT scan evidence of abnormally dilated bronchi.^27^ HRCT scans are not routine and only undertaken at the discretion of the attending physician.

#### CSLD

Chronic respiratory symptoms/signs of bronchiectasis but where either a chest HRCT scan has not been done or not shown radiographic evidence for this diagnosis.^19 31^

#### Treatment failure

If there are six community-treated ARLIs or three ALRI hospitalisations during the 12-month intervention (or prorata for those on 6 months). These are captured on the child’s electronic records as done previously.^19^ Where possible, participants will be offered a referral to either a paediatric respiratory specialist or paediatrician.

### Sample collection

Upper airway respiratory pathogen carriage and antibiotic resistance will be assessed using nasopharyngeal swabs in accordance with the WHO recommendations for pneumococcal carriage studies.^32^ At the end of the study, Australian and New Zealand samples will be tested at Menzies and Timorese samples tested at the Timor-Leste National laboratory using standardised culture protocols across all sites. Nasopharyngeal swabs will be thawed and cultured for *Streptococcus pneumoniae*, *H. influenzae*, *Moraxella catarrhalis* and *Staphylococcus aureus*. Antibiotic susceptibility of *S. pneumoniae*, *H. influenzae*, *M. catarrhalis* and *S. aureus* isolates will be determined by the European Committee on Antimicrobial Susceptibility Testing (EUCAST) disc diffusion method.^23^ The minimum inhibitory concentration for azithromycin will be determined by E-tests where non-susceptibility is identified by disc diffusion.^23^ EUCAST criteria will be used to define non-susceptibility, as done previously.^14 19 23^

### Future Microbiological studies

Microbiota analysis of nasopharyngeal swabs will be undertaken when funding becomes available. Where possible, oropharyngeal swabs and faecal samples (OMNIgene, DNA Genotek) for microbiota analyses will also be collected at baseline, 12 (or 6 months if opting out of trial medication at 6 months), 18 and 24 months and will be stored for future testing.

### Outcomes

The primary outcome is the annualised rate of medically attended ALRIs during the intervention period of 6–12 months when the participants received the trial medications. Outcomes are assessed at 6 and 12 months for those who received 6 and 12 months of study trial medication, respectively.

Secondary outcomes are the rates and proportions of children with:

1. ALRI hospitalisations during the intervention period at 6–12 months.
2. ALRI hospitalisations during and post-intervention (at 24 months post-commencement of trial medications).
3. Adverse events (eg, nausea, vomiting, diarrhoea and rash).
4. Nasopharyngeal colonisation by macrolide-resistant respiratory pathogens at baseline, (6), 12, 18 and 24 months.
5. Chronic respiratory symptoms/signs suggestive of underlying CSLD or bronchiectasis at 24 months.
6. ICER on the potential benefits (in terms of savings in hospitalisations and medical evacuations, and other associated costs to health services and patients) of azithromycin compared with placebo over the additional costs of administering the drug.

### Data monitoring, management and analyses

All study participants are assigned a unique study identifier at enrolment, which is the primary identifier on all study records including bio-specimen labels throughout the trial. Confidentiality is maintained in accordance with GCP guidelines and with respective HREC requirements at each site. All identifiable data from study participants are retained on a password-protected REDCap database and in locked filing cabinets at each study site. Each site is responsible for data entry. Data management is overseen by the principal investigator (GBM) at Menzies. Access to these data is only provided to immediate members of the research team except if requested by legislative or regulatory agencies, or by the respective HREC at each site.

All adverse and serious adverse events are monitored by an iDMC which was established prior to commencing the trial. Reference groups at each site oversee cultural aspects of the RCT.

A detailed statistical analysis plan will be prepared and approved by the chief investigators and the iDMC prior to undertaking the final analysis. Data will be audited, cleaned and locked before any analyses are undertaken. The investigators will only see analyses of pooled data until the statistical analysis code has been written (on pooled data) and agreed, and the trial has terminated. At this point, unblinding of investigators will occur. The analysis will be directed by a biostatistician. Results will be reported in accordance with the Consolidated Standards of Reporting Trials guidelines. We will use an ‘intention-to-treat’ approach for the primary analysis. Missing data will not be imputed. No interim analysis is planned. Any post hoc analysis or unplanned analyses will be described clearly.

#### Primary aim

To determine the efficacy of azithromycin, the annualised rate of medically attended ALRIs in those randomised to receive 6–12 months of azithromycin will be compared with those receiving placebo (controls). A negative binomial regression model and incident rate ratio (IRR) 95% CI, including treatment group and the number of months taking treatment in the study included as an offset, will be used to determine between-group differences with 95% CIs. Subgroup analyses will be done for age (≤12, >12 months) and adherence (≤ or >70%).

#### Secondary aims

##### Clinical outcomes

Determine the efficacy of 6–12 months of weekly azithromycin (compared with placebo) on (1) rates and proportion of children with ALRI hospitalisations between treatment groups using negative binomial regression (reporting IRR, 95% CI) while taking the intervention; (2) rates and proportions of children with ALRI hospitalisations between treatment groups using negative binomial regression (reporting IRR, 95% CI) during and post-intervention (at 24 months postcommencement of trial medications); (3) adverse events will be compared between treatment groups using risk differences (95% CI) while on the intervention; and (4) the proportion of children with chronic respiratory symptoms/signs suggestive of underlying CSLD or bronchiectasis at 24 months between treatment groups.

##### Laboratory outcomes

Determine the efficacy of 6–12 months of weekly azithromycin (compared with placebo) on nasopharyngeal respiratory pathogen carriage and antimicrobial resistance using longitudinal analysis and logistic regression, as done previously.^19^

##### Cost-effectiveness

ICERs using the Bootstrap method for inference will be used. Cost estimation will involve azithromycin, medical retrieval and patient/escort travel costs by using actual expenditure and contractual data and costing for hospitalisation by using average cost estimates per Australian Refined Diagnosis Related Groups. A 6–12-month delay is expected if actual expenditure and costing data are adopted for analysis (rather than a cost model based on historical information). Study-specific visit/procedures (ie, medication administration) will be identified in the analysis and adjusted accordingly (ie, removed from cost model data). The health economic analyses will be reported in a separate manuscript.

##### Sample size

Based on our primary outcome (rate of medically attended ALRI), 80 children per group (total sample size n=160, including 15% attrition) will provide 90% power to detect a 40% reduction (IRR=0.6) in medically attended ALRI episodes per child-year. We assumed a rate of three medically attended ALRI episodes per child-year in the control group, based on community presentations for respiratory illnesses in our setting,^33^ a shape parameter of k=0.4, and a two-sided significance level of 5%. Children who are withdrawn or lost to follow-up (at 6–12 months) will be replaced until the required sample size is obtained. Our previous bronchiectasis RCT^19^ reported a halving of respiratory exacerbations in the azithromycin group and the maximal benefit was achieved between 4 and 14 months of the intervention.^34^

##### Trial oversight

The iDMC meet every 3–6 months. Meeting frequency is deemed by the committee members. This trial is overseen by the chief investigators of the grant (listed below). It is sponsored and monitored by Menzies (Darwin, Australia).

### Ethics and dissemination

The Northern Territory Department of Health and Menzies (HREC 2019-3401); Health and Disability Ethics Committee, New Zealand (19/STH/108); and the Institute National of Health-Research Technical Committee, Timor-Leste (1901 MS-INS/GDE/IX/2022), approved the study.

The RCT is conducted in accordance with Australia, New Zealand and Timor-Leste legislation, and the Australian NHMRC guidelines for Ethical Conduct of Research, including that for First Nations Australians. The results will be communicated in aggregated format to study participants, their families and respective communities via written and oral presentations as appropriate to the local setting. We will publish the trial results in peer-reviewed medical journals and share with the medical and academic community at relevant national and international conferences, via reports to funding bodies, and reports and/or presentations to other relevant stakeholder groups (eg, First Nations Reference Groups) and policy makers (site government authorities, Therapeutic Goods Administration). Appropriate authorship guidelines will be used. We will not use professional writers.

## Discussion

Despite the high burden and long-term consequences of severe ALRIs, clinically feasible interventions (other than vaccine strategies) to prevent recurrent ALRI hospitalisations in early childhood are limited. Evidence-based strategies to reduce ALRIs, prevent CSLD and bronchiectasis, and preserve lung function are required. We previously found weekly azithromycin for 12–24 months halved the frequency of respiratory exacerbations in First Nations children with CSLD or bronchiectasis.^19^ It was also well tolerated and accepted by remote communities. Whether this is effective at preventing recurrent ALRIs in children without bronchiectasis who are deemed to be an increased risk of chronic lung disease is unknown. While there is some evidence that intermittent short courses of azithromycin prevent wheezing episodes in non-First Nations children,^35^,^37^ this is not a universal finding^38^ and none involved populations at high risk of developing CSLD or bronchiectasis. Despite limited evidence, clinicians in some settings, including the Northern Territory of Australia, frequently prescribe long-term azithromycin in children with recurrent ALRIs, but who lack underlying bronchiectasis. High-level evidence is needed to justify this practice given the risks of increasing antimicrobial resistance, safety and healthcare costs have not been examined. Our RCT will address these knowledge gaps in children at high risk of recurrent ALRIs and subsequent CSLD or bronchiectasis.

### Choice of duration of intervention

We chose two different durations of intervention (ie, 6 or 12 months of azithromycin/placebo) for two main reasons. First, we found previously the peak effect of using azithromycin for preventing bronchiectasis exacerbations occurred between 17 and 62 weeks^34^; thus, an intervention >4 months is justified. Second. while it would have been ideal to use a single intervention for 12 months, some parents/guardians declined to participate because they considered the intervention was too long. Thus, after recruiting five children, we altered the protocol so parents could choose either 6-month or 12-month intervention. Nevertheless, to date, just 2% of participants have opted out at the 6-month time-point.

### Choice of study outcomes

ALRIs were chosen as the primary outcome since early and recurrent ALRIs are associated with impaired lung health,^6^ future reduced lung function^39^ and subsequent increased risk of chronic lung diseases (eg, CSLD, bronchiectasis and COPD)^9 10 40^ in the communities from which the study population is drawn. Furthermore, ALRIs in young First Nations children are the leading causes of hospitalisation,^41 42^ and persistent symptoms post-hospitalisation,^14 17^ including readmission for a respiratory illness within the subsequent 6 months.^14^ ALRIs are defined as per our previous RCTs.^14 28^

Secondary outcomes (ALRI hospitalisations, children with chronic respiratory symptoms/signs suggesting underlying CSLD or bronchiectasis, adverse events, carriage and antimicrobial resistance in upper airway respiratory pathogens, and cost-effectiveness) were chosen because of their importance to clinicians and consumers.^43^

### Patient and public involvement

20 parents/guardians of children admitted to the Darwin hospital with an ALRI were involved in preliminary discussions on the trial design. 16 (80%) parents/guardians expressed their support for their child to take long-term medicine to prevent further ALRIs. We will directly assess the burden of parents/guardian’s participation in this study at the final visit questionnaire, about perceptions of the trial, future directions and how involvement in studies impacts participants/families. We plan to disseminate trial outcomes to study participants through plain language statements and based on responses in the final visit questionnaire.

Public involvement included the trial being reviewed and endorsed by First Nations groups at each site prior to commencing participant recruitment. These groups provided advice to ensure the study design was culturally appropriate to local contexts. Ongoing updates to these groups throughout the trial will be undertaken. We will disseminate study results to the wider community through this publication, future workshops and education sessions to health professionals.

### Potential study limitations

First, heterogeneity exists between study sites but is reduced by including local community workers and having strict protocols for inclusion/exclusion criteria, data collection and measuring study outcomes. Further, randomisation is stratified, thus reducing the impact of any heterogeneity between sites. Second, directly observed medication adherence may not be possible in all participants. However, research or community health staff visit families weekly to dispense medications, and/or use medication logs with frequent phone contact, which should reduce the risk of non-adherence. Third, relying upon reviewing medical records risks missing some ALRIs if these records are incomplete.

### Impact of COVID-19

Recruitment commenced in October 2020; however, this was seriously impacted by the COIVD-19 pandemic due to strict government and organisational lockdowns during 2021–2022, which meant we were unable to enter site hospitals or follow-up children in their communities. When lockdown restrictions eased in late 2022, additional restrictions from local site hospitals and research institutions further impacted recruitment and participant follow-up. After restrictions eased, recruitment increased; however, families were COVID-weary, and some were reluctant to participate in RCTs in the early post-pandemic period. We therefore initiated mitigation steps that included medication dispensing and follow-up community by local health staff. Nevertheless, we are uncertain if our study findings will be adversely affected by the public health response to the pandemic.

### Summary

Here, we describe our parallel, double-blind, placebo-controlled multicentre, international RCT that aims to determine the efficacy of 6–12 months of azithromycin at improving clinical outcomes among children aged <2 years posthospitalisation for an ALRI and who are from communities and populations at high risk of chronic lung disease.

## Regulatory

### Registration

The ‘PETAL’ trial is approved by the Therapeutic Goods of Australia (CT-2019-CTN-00 958-1) and is registered with the Australian and New Zealand Clinical Trial Registry: https://www.anzctr.org.au/Trial/Registration/TrialReview.aspx?id=377132&isReview=true (ACTRN12619000456156).

### Governance

Site hospital approval for the ‘PETAL’ trial was obtained prior to trial commencement.

## Dissemination

### Access to data

The dataset will be held by the trial sponsor, Menzies, Northern Territory, Australia. The principal investigator and study statistician will have access to all study data at the completion of the trial. There are no plans to grant public access to the full protocol, participant-level dataset or statistical code due to cultural considerations such as including respect for privacy, sensitivity to local norms/traditions, and potential implications for community relations.

## supplementary material

10.1136/bmjopen-2024-097455 online supplemental file 1
