# Supplementary material for: Azithromycin to prevent acute lower respiratory infections among Australian and New Zealand First Nations and Timorese children (PETAL trial): study protocol for a multicentre, international, double-blind, randomised controlled trial
Source: BMJ Open. 2025 Feb 5;15(2):e097455. doi: 10.1136/bmjopen-2024-097455 (PMC11800299; doi:10.1136/bmjopen-2024-097455)
Supplement: online supplemental file 1 [file bmjopen-15-2-s001.docx]

**SUPPLEMENT**

**STUDY ID**

**Preventing rEcurrenT Acute Lower respiratory infections (ALRI) in young Indigenous children using long-Term, once-weekly Azithromycin: A multi-centre randomized controlled trial**

**(PETAL) Study**

**“This Means You Can Say No”**

**CONSENT FORM**

I have read/discussed the information booklet and understand that participation is voluntary and that I can withdraw from the study at any time, without giving a reason and it won’t affect the medical care my child receives.

I give permission for my child to have a respiratory check-up at enrolment (in hospital), and again at 1, 4, 8, 12, 18, and 24 months by the research team.

I will complete a medical history and questions relating to my child’s lung health at each visit.

I give permission for my child’s medical records to be reviewed throughout the study (hospital, health clinic, general practitioner, immunisation database) for illnesses related to my child’s health.

The results obtained from this study may benefit my child’s medical management, but will not result in any extra medical costs to me.

The results of this research will be made accessible to the wider medical community but my child’s involvement and identity will not be revealed.

I,___________________________________________________________________________

^(full name of parent/guardian)^

Being the______________________________ of____________________________________

^(state relationship e.g. Parent, guardian etc) (full name of child)^

of______________________________________,_____________________, _____, ________

^(street address) (suburb/town) (state) (postcode)^

I give consent to deep nasal swabs (NPS) _(in hospital, and at 12, 18 and 24 months check-up (where able))_ Yes No

I give consent to throat swabs (OPS) _(in hospital, and at 12, 18 and 24 months check-up (where able))_ Yes No

I give consent to stool (poo) samples _(in hospital, and at 12, 18 and 24 months check-up (where able))_ Yes No

I give consent to the researchers to include my child’s de-identified data (NPS/OPS/poo) in journals who request inclusion of de-identified datasets.

Yes No

I choose for the following to happen to the collected specimens (NPS/OPS/poo) (please tick the relevant box)

**When the research project is finished, I would like the specimens collected from ‘my child’**

1. To be frozen and used for further research about lung health in children.

*This will be done in the Menzies laboratory or, if necessary, in another laboratory in Australia or internationally that is approved by Menzies. Any further research will be subject to approval by the relevant ethics committee*

**OR**

1. The specimens (swabs/stool) are to be destroyed and never again used after this study

I give consent to be interviewed at the 24-month visit, and for my de-identified data to be used towards planning of future clinical research trials.

Yes No

I give consent to have my interview audio recorded Yes No

I have had any questions or queries answered to my satisfaction and I have been informed that confidentiality of the information will be maintained and safeguarded.

**Name of parent or guardian:** ____________________________________________________

**Signature of parent or guardian:** ____________________________**Date** _____/_____/_____

**Name of independent witness:** __________________________________________________

**Signature of independent witness**: _________________________ **Date** _____/_____/_____

**Researcher’s name**: ___________________________________________________________

**Signature of researcher**: __________________________________**Date** _____/_____/_____

**Interpreter’s name (if necessary):** _______________________________________________

**Signature of Interpreter (if necessary):** ______________________**Date** _____/_____/_____
